# Shape-Memory-Recovery Characteristics of Microcellular Foamed Thermoplastic Polyurethane

**DOI:** 10.3390/polym12020351

**Published:** 2020-02-06

**Authors:** Chang-Seok Yun, Joo Seong Sohn, Sung Woon Cha

**Affiliations:** School of Mechanical Engineering, Yonsei University, 50, Yonsei-ro, Seodaemun-gu, Seoul 03722, Korea; changseok2614@yonsei.ac.kr (C.-S.Y.); ssamjjang87@yonsei.ac.kr (J.S.S.)

**Keywords:** shape-memory polymer, shape-memory-recovery characteristics, thermoplastic polyurethane, microcellular foaming process, injection-molding process, batch process

## Abstract

We investigated the shape-recovery characteristics of thermoplastic polyurethane (TPU) with a microcellular foaming process (MCP). Additionally, we investigated the correlation between changes in the microstructure and the shape-recovery characteristics of the polymers. TPU was selected as the base material, and the shape-recovery characteristics were confirmed using a universal testing machine, by manufacturing dog-bone-type injection-molded specimens. TPUs are reticular polymers with both soft and hard segments. In this study, we investigated the shape-memory mechanism of foamed polymers by maximizing the shape-memory properties of these polymers through a physical foaming process. Toward this end, TPU specimens were prepared by varying the gas pressure, foaming temperature, and type of foaming gas in the batch MCP. The effects of internal structural changes were investigated. These experimental variables affected the microstructure and shape-recovery characteristics of the foamed polymer. The generated cell density changed, which affected the shape-recovery characteristics. In general, a higher cell density corresponded to a higher shape-recovery ratio.

## 1. Introduction

The objective of this study was to investigate the changes in the shape-memory-recovery characteristics of thermoplastic polyurethane (TPU), with changes in the microstructure by employing a microcellular foaming process (MCP), which is a physical foaming process [1,2,3,4]. The MCP, which is applied to TPU as a matrix, is a method for producing microsized cells having a density of >10^9^ cells/cm^3^ within 10 μm inside polymer plastics. This method was recently applied to the production of advanced electronic products [5]. The plastic-manufacturing method with fine cells has several advantages, such as reduced production time, reduced product weight, reduced material cost, minimized post-transformation, insulation, and soundproofing.

In this study, a batch MCP was used. In a pressure vessel at a high temperature and high pressure, the outside of the polymer plastic was under a relatively high pressure compared with the inside. The dissolved gas was present in a single phase in the polymer, and nuclei were created in the polymer through phase changes owing to the thermodynamic instability caused by removing the pressure conditions. At this time, if heat were applied above the glass transition temperature of the polymer, the cells would grow. The matrix polyurethane consisted of two physical structures: a soft segment and a hard segment. In general, the crystalline phase within the soft-phase matrix forms a discontinuous region owing to van der Waals forces and hydrogen bonds [6].

It is considered that by applying the MCP, it is possible to expand the area of the soft phase by increasing the distance between the crosslinking points corresponding to the crystalline phase, using the uniform fine cells formed in the polymer matrix. The soft segment plays an elastic role in deformation and recovery and is considered to have excellent recovery performance by maximizing the recovery deviation for the programmed stimulus, as it has the freedom to move. By applying an MCP, uniform cells are formed in the polymer matrix, which is not biased according to the orientation of the applied stimulus and can have uniform isotropy with regard to the recovery performance. Additionally, because the MCP has advantages such as light weight, dimensional stability, and moldability improvement, combining it with a shape-memory polymer can yield a new direction. Thus far, research on shape-memory polymers have primarily been focused on the study of shape-memory-recovery characteristics via extension polymerization and modification or the addition of fillers to side chains. There have been a few studies in which the shape-memory-recovery characteristics have been investigated by applying a physical foaming process [7,8,9,10,11]. In this study, we attempted to optimize the shape-recovery characteristics by changing the physical properties without performing additional polymerization and reformation or incurring large costs.

In general, foamed polymer materials are lighter compared with unfoamed materials, and they have excellent thermal insulation owing to their internal fine cells. However, foamed polymer materials may exhibit lower mechanical strength than unfoamed materials [12]. With the addition of a filler to solve this problem, the mechanical strength of the material is improved, but the shape-memory ability may be deteriorated. When the MCP is applied, good shape-memory performance, low weight, insulation, sound insulation, and dimensional stability can be realized simultaneously while minimizing the mechanical-strength degradation of the material, by forming fine and uniform cells. In the future, we expect to develop light and heat-resistant shape-memory polymers with a high shape-memory-recovery rate compared with unfoamed shape-memory polymers. Such polymers can be applied in various fields, such as smart clothing, shape-memory fibers, and hinges, and have several potential applications [13,14,15,16,17]. For example, when applied to a shape-memory stitching thread where a knot is formed beyond a certain temperature, the mechanical strength is similar to that of a conventional unfoamed material, and the density is low; thus, the physical burden on the patient can be reduced [18,19,20,21,22,23,24]. Additionally, for clothing materials that recover their shape while resting at a certain temperature, consumer convenience can be enhanced by reducing the recovery time. It is anticipated that by combining the MCP of this study with existing unfoamed shape-memory composite hinges, it will be possible to produce materials with insulation and soundproofing characteristics [25,26,27,28,29,30].

## 2. Materials and Methods

### 2.1. Materials

#### TPU

Shape-memory polymers must have excellent durability against repetition because they are fixed and recovered in shape. Additionally, owing to the difference in the mobility of the molecular chains around the phase-transition temperature of the polymer, such as the glass transition temperature or crystalline melting temperature, the polymer should have the characteristics of shape recovery and shape fixation. Thus, in this study, TPU (BASF Corporation, Ludwigshafen, Germany, Product Grade No. Elastollan 1175AW, density = 1.14 g/cm^3^, glass transition temperature = −48 °C) was selected as the matrix material, which had a spherical pellet shape, similar to a polymer resin. The TPU used in this study has a value of 20–50 g/10 min in melt flow index (MFI) conditions (190 °C, 10 kg) according to procedure B of ASTM D 1238 and is suitable for use in injection and extrusion processes. The TPU was applied to the injection-molding process to prepare a specimen for investigating the shape-memory-recovery characteristics.

### 2.2. Methods

#### 2.2.1. Injection-Molding Process

An injection-molding process was performed to evaluate the shape-memory characteristics of microcellular foamed TPU. The screw mounted on the injection-molding machine (WOOJIN SELEX Corp., Korea, Boeun, Product No. E-120) had a length-to-diameter ratio of 28:1 and a clamping force of 120 tons. Tensile specimens corresponding to the test specification D638 were prepared to measure the shape-recovery properties using American Society for Testing and Materials standard molds. The heaters of the injection molding machine were installed in six places, and the temperature was set from the nozzle from which the molten polymer was discharged to the barrel heater (the number of partial heaters mounted on the barrel increased as they moved away from the nozzle). The process temperature was set as 180 °C, and the process conditions are presented in detail in Table 1. However, the mechanical properties were not measured using the standard specimen; a dog-bone-type specimen was used only to investigate the shape-memory-recovery characteristics.

#### 2.2.2. Batch MCP

**Figure 1:**
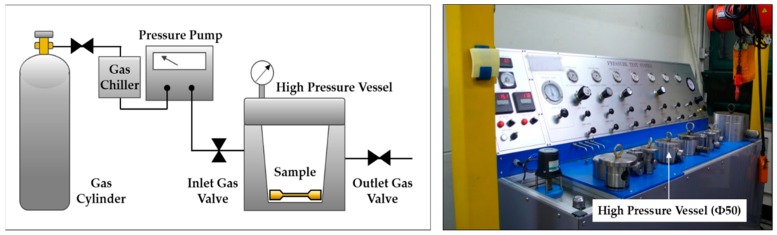
Batch microcellular foaming process (MCP) adopted in this study (**left**) and batch processing equipment used in the experiment (**right**).

Inert gases can be dissolved in the polymer when the pressure of the gases is increased. This process is called a microcellular batch process (Figure 1). In the microcellular batch process, solid-state polymer specimens are placed inside a high-pressure vessel. Then, a high-pressure inert gas is injected to dissolve the gas into the polymer due to the partial pressure difference. The gas dissolved in the specimens is heated at atmospheric pressure and escapes into the atmosphere, forming dense cells inside of polymer.

In the microcellular batch process, the gas species, gas pressure, and foaming temperature are influenced by the cell nucleation in the polymer. Thus, experiments were performed in which the gas pressure, foaming temperature, and gas species were controlled as variables. The experimental conditions for the preparation of the foamed TPU specimens are presented in Table 2, Table 3 and Table 4.

As shown in Table 2, the foaming-gas pressures were set as 3, 4, and 5 MPa. We examined the correlation between the shape-recovery characteristics and the foaming-gas pressure of the foamed specimens. As shown in Table 3, the foaming temperatures were set as 50, 100, and 150 °C. The foaming temperature affected the cell density and, consequently, the shape-recovery characteristics. Finally, carbon dioxide, nitrogen, and helium, which are the main gases used in the microcellular batch process, were used as the blowing gases. The foaming properties changed with respect to the gas species, and the effects of the gas species on the shape-recovery characteristics of the foamed TPU were investigated.

#### 2.2.3. Shape-Recovery Experiment

**Figure 2:**
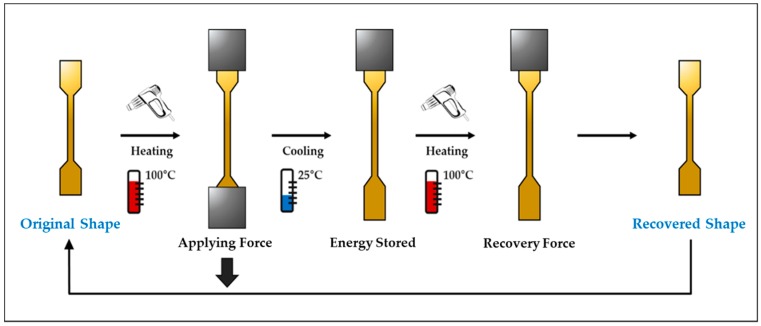
Experimental method employed in this study.

In this study, shape-recovery experiments were conducted using foamed TPU specimens. For the shape-recovery test, a universal testing machine (Qmesys, Korea, Anyang, Product No. QM100) was used to apply external force uniformly, and the specimens were heated at 100 °C to provide the energy required for shape deformation. The heating time, cooling time, and reheating time were all 5 min (Table 5). In Figure 2, the original shape specimens were applied to heat (100 °C) to provide the energy needed for shape deformation. The specimens were deformed by applying a uniform external force using a universal testing machine. Then, the deformed specimens were cooled to room temperature (25 °C) and re-heated to confirm the extent of their shape recovery. In this process, the external force did not work. After heating, recovery ratio was calculated by recovered shape of specimens. The tensile lengths of the specimens were set as 50 mm, and the tensile speed was 10 mm/min. The displacement was examined in the plastic-deformation region. By setting the displacement of the specimen to the plastic-deformation region, the effects of the cells in the specimen on the shape-recovery characteristics were examined.

## 3. Results and Discussion

### 3.1. Changes in Shape-Recovery Characteristics According to Foaming Gases

**Figure 3:**
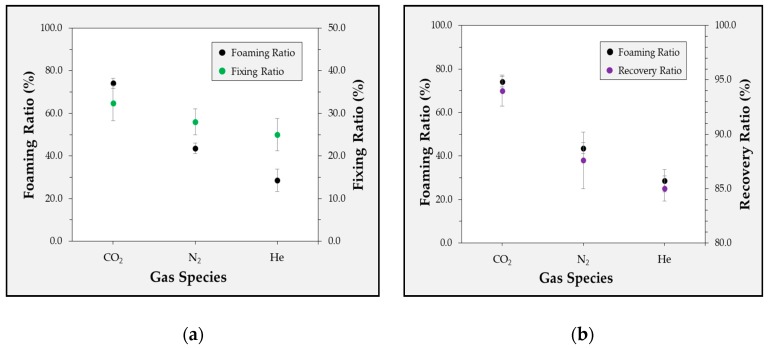
Experimental results for the correlation between the foaming ratio and the fixing ratio (**a**) and recovery ratio (**b**) according to the gas species.

**Figure 4:**
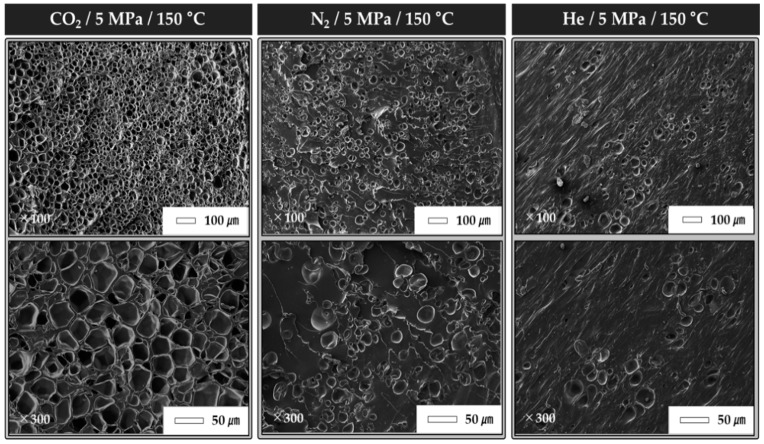
Cross-sectional SEM images of TPU specimens for different gas species.

Figure 3 shows the changes in the shape-recovery characteristics according to the gas species and foaming ratio. The foaming ratio was 74.1% in CO_2_, 43.6% in N_2_, and 28% in He, and the shape-recovery characteristics differed accordingly. The shape-fixing ratio was 32.4% in CO_2_, 28.0% in N_2_, and 22.0% in He. The shape-recovery ratio was 94% in CO_2_, 86.7% in N_2_, and 85% in He. Thus, the shape-recovery ratio and shape-fixing ratio improved as the foaming ratio of the specimens increased. Scanning electron microscopy (SEM, JEOL, Korea, Seoul, Product No. IT-500HR) images were used to analyze the foaming status of the specimens according to the gas species. Cross-sectional images of the specimens for the different gas species are shown in Figure 4. The number of cells in the specimen differed significantly according to the gas species. In addition to the foaming ratio of the specimens, the foaming status was confirmed by checking the number of cells. Here, N_C_ represents the number of cells in the SEM image; D_1_ represents the density of the polymer before the foaming process; and D_2_ represents the density of the polymer after the foaming process.

Number of cells = N_c_ × (D_1_/D_2_)^1/3^ (1)

Table 6 presents the number of cells for each experimental condition. The number of cells was calculated using the SEM images and Equation (1). The largest number of cells was 62.8 (in CO_2_), followed by 30.4 in N_2_ and 22.2 in He. The experimental results indicated that the foaming ratio increased as the number of cells increased. Additionally, it is considered that the increased foaming ratio influenced the shape-recovery characteristics.

### 3.2. Changes in Shape-Recovery Characteristics According to Gas Pressure

**Figure 5:**
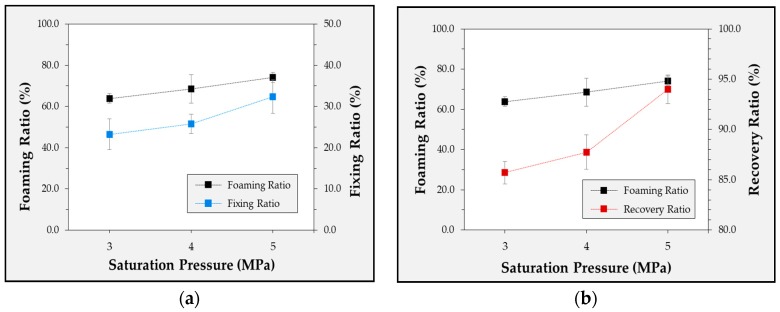
Experimental results for the correlation between the foaming ratio and the fixing ratio (**a**) and recovery ratio (**b**) according to the saturation pressure.

**Figure 6:**
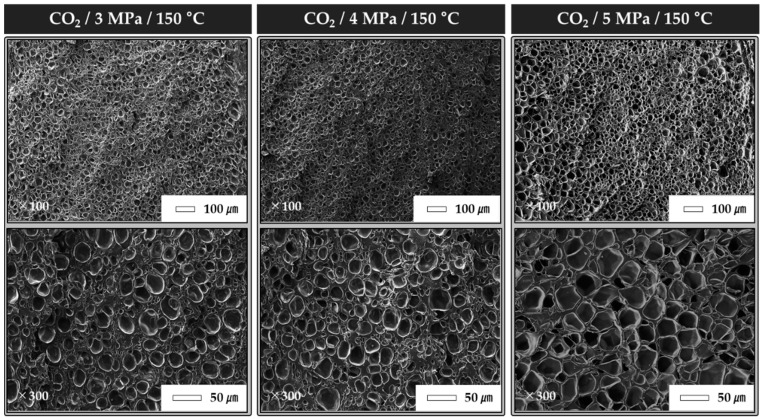
Cross-sectional SEM images of TPU specimens at different saturation pressures.

According to the experimental conditions used in this study, the CO_2_ condition yielded the best shape-recovery characteristics. In the case of foaming ratio, CO_2_ showed the highest value, followed by N_2_ and He. As gas pressure increased, foaming ratio and cell density increased. The results of this study confirmed that foaming does not proceed as well in N_2_ and He as it does in CO_2_. Therefore, in this study, the pressure conditions of 5 MPa were compared to confirm the difference according to the gas species. In addition, it was carried out under CO_2_ conditions to confirm the change resulting from the different pressure conditions. Therefore, in this experiment, the foaming gas was CO_2_, and the gas pressures were set as 3, 4, and 5 MPa. The shape-recovery characteristics of the specimens were investigated with respect to the foaming gas pressures. Figure 5 shows a comparison of the shape-recovery characteristics and foaming ratio with different foaming-gas pressures. The highest shape-recovery ratio was observed at 5 MPa. At 3 MPa, the shape-fixing ratio was 23.3%, and the shape-recovery ratio was 85.7%; the shape-recovery characteristics were worse than those at 4 and 5 MPa. As shown in Table 7 and Figure 6, the foaming ratio of TPU under gas pressures was higher than that under other experimental conditions. The foaming ratio was 63.9% at 3 MPa, 68.5% at 4 MPa, and 74.1% at 5 MPa. Additionally, the number of cells calculated using the SEM image was 47.4 at 3 MPa, 54.6 at 4 MPa, and 62.3 at 5 MPa. The results indicated that a higher foaming ratio of the specimens corresponded to better shape-recovery characteristics. In particular, we assumed that the shape-recovery characteristics of the foamed TPU specimens improved as the number of cells per unit length increased.

### 3.3. Changes in Shape-Recovery Characteristics According to Foaming Temperatures

**Figure 7:**
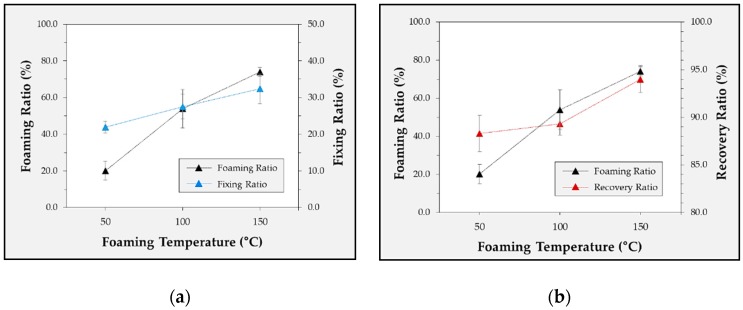
Experimental results for the correlation between the foaming ratio and fixing ratio (**a**) and recovery ratio (**b**) according to the foaming temperature.

**Figure 8:**
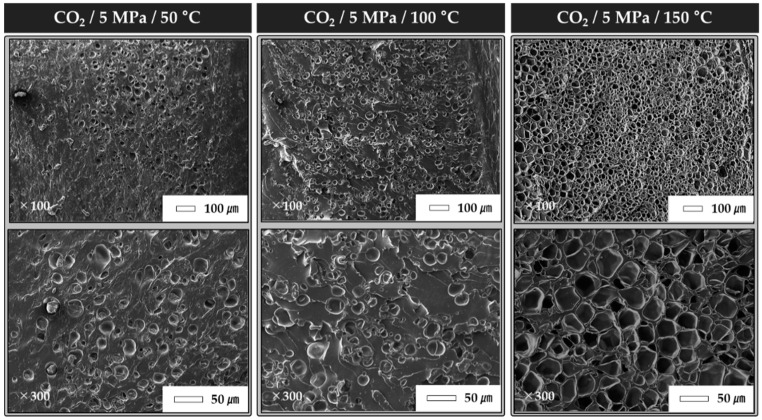
Cross-sectional SEM images of TPU specimens at different foaming temperatures.

The foregoing results confirm that the highest foaming ratio and best shape-recovery characteristics were achieved using CO_2_ at 5 MPa. In this experiment, we examined the shape-recovery characteristics with respect to the foaming temperature by controlling the foaming temperature. The foaming temperatures were set as 50, 100, and 150 °C. CO_2_ was used as the foaming gas, and the gas pressure was set as 5 MPa. The blowing gas and gas pressure were set according to the conditions that yielded the best characteristics in the previous experiment. Figure 7 shows the shape-recovery characteristics of foamed TPU specimens with different foaming temperatures. At 50 °C, the foaming ratio was 17.4%, which was the second-lowest value (after the He condition). The shape-fixing ratio was 23%, and the shape-recovery ratio was 86%. At 100 °C, the foaming ratio was 49.4%; the shape-fixing ratio was 27.5%; and the recovery ratio was 89.3%. As shown in Table 8 and Figure 8, the foaming ratio was significantly affected by the foaming temperature. The number of cells increased linearly with an increase in the foaming temperature. In this experiment, the best highest shape-recovery characteristics were obtained at 150 °C. Even when a similar amount of gas was dissolved through the microcellular batch process, the foaming status was changed according to the foaming temperature. Additionally, it was considered that the shape-recovery characteristics were improved as the foam became uniform inside the polymer.

## 4. Conclusions

We investigated the shape-recovery characteristics of TPU with the MCP. Additionally, we examined the correlation between changes in the microstructure and the shape-recovery characteristics of the polymers. Experiments were performed to determine the variation in the shape-recovery ratio of the MCP for the TPU according to the foaming conditions, and the results were as follows.

CO_2_, N_2_, and He were applied as blowing gases to perform a batch process. The prepared specimens exhibited higher shape-recovery ratios than the neat specimen. The He condition yielded a 7.7% improvement in the shape-recovery ratio compared with the neat specimen. This was the lowest shape-recovery ratio among those obtained for all the experimental conditions. Under the same gas pressure, the CO_2_ condition yielded the highest foaming ratio, and N_2_ and He yielded relatively low foaming ratios. It can be considered that the highest shape-recovery ratio was obtained under a high foaming ratio. The shape-fixing ratios for all the experimental conditions were lower than that of the neat specimen. For example, the difference between the shape-fixing ratio under the 3 MPa condition with a foaming ratio of 63.9% and under the He condition with a foaming ratio of 14.8% was 1%. Therefore, we assumed that the foaming ratio had a relatively small effect on the shape-fixing ratio, compared with its effect on the shape-recovery ratio.

The foaming characteristics of TPU were affected by the gas pressure. In this case, we used CO_2_ as a blowing agent because CO_2_ was foamed at various pressure ranges. On the other hand, N_2_ and He did not foam below 5 MPa. Therefore, we confirmed the following results when using CO_2_ as the blowing agent: as the foaming ratio increased the shape-recovery ratio and shape-fixing ratio increased. The shape-recovery ratio was 8.6% higher than that of the neat specimen at 3 MPa and 19.1% higher at 5 MPa. In the case of the shape-fixing ratio, reductions of 35.6%, 28.7%, and 10.5% were observed at 3, 4, and 5 MPa, respectively, compared with the neat specimen. The foaming ratio was >60% in the range of 3–5 MPa. As shown in Table 6, the number of cells per unit length was the largest at 5 MPa. The shape-recovery ratio and shape-fixing ratio improved as the number of cells increased, according to the pressure condition. Thus, it is considered that the number of cells affects the improvement in the shape-recovery ratio and shape-fixing ratio of TPU.

Experiments were performed in which foaming temperatures of 50, 100, and 150 °C at 5 MPa were used; this yielded the best shape-memory-recovery characteristics among the gas-pressure conditions. Additionally, CO_2_ was used as a blowing agent, which had the best shape-memory-recovery characteristics among the gas species. In the case of the shape-fixing ratio, reductions of 36.5%, 24%, and 10.5% were achieved at 50, 100, and 150 °C, respectively. In the case of the shape-recovery rate, improvements of 9%, 13.2%, and 19.1% were observed at 50, 100, and 150 °C, respectively. By varying the foaming temperature, it was confirmed that for the same gas pressure, the foaming ratio varied with the amount of gas dissolved in the polymer. The shape-memory-recovery characteristics also appeared to be affected by the foaming ratio of the TPU, which varied with the foaming temperature. The maximum number of cells was observed at 150 °C, and the shape-recovery ratio changed with changes in the cell density due to cell nucleation.

In studies about polymer foams, epoxy-based foams were used, and nanometer sized additives were added along with the polymer foams. These studies were done to investigate the change of shape-memory-recovery characteristics according to foam formation and additive contents. It was found that the shape-memory-recovery properties of the polymer composites decreased as the additives content increased. However, we investigated the relationship between microstructures and shape-memory-recovery characteristics. MCPs were applied to change the microstructure of TPU, and it was confirmed that the generated cell size was set smaller than that of the conventional foaming process by chemical synthesis. MCPs, physical foaming processes, and the use of an inert gas were different from the conventional chemical foaming, and as the cell distribution increased, the shape recovery characteristics were improved.

## Figures and Tables

**Table 1:** Detailed experimental conditions of the injection-molding process for specimen preparation.

Parameter	Experimental Conditions					
Injection temperature (°C)	Nozzle	H1	H2	H3	H4	H5
	180	170	160	150	140	130
Injection pressure (MPa)	8					
Injection time (s)	3					
Holding pressure (MPa)	5					
Holding time (s)	9					
Cooling time (s)	100					

**Table 2:** Batch-process conditions with different foaming gases.

Parameter	Experimental Conditions
Foaming gas	CO_2_, N_2_, He
Gas pressure (MPa)	5
Saturation time (h)	48
Foaming temperature (°C)	150

**Table 3:** Batch-process conditions with different gas pressure.

Parameter	Experimental Conditions
Foaming gas	CO_2_
Gas pressure (MPa)	3, 4, 5
Saturation time (h)	48
Foaming temperature (°C)	150

**Table 4:** Batch-process conditions with different foaming temperatures.

Parameter	Experimental Conditions
Foaming gas	CO_2_
Gas pressure (MPa)	5
Saturation time (h)	48
Foaming temperature (°C)	50, 100, 150

**Table 5:** Detailed experimental conditions for investigating the shape-recovery characteristics.

Parameter	Experimental Conditions
Heating temperature (°C)	100
Heating time (min)	5
Cooling time (min)	5
Displacements (mm)	50
Speed (mm/min)	10

**Table 6:** Foaming properties of thermoplastic polyurethanes (TPU) according to gas species.

Experimental Conditions			Number of Cells	Foaming Ratio (%)
Species of Gas	Pressure (MPa)	Temperature (°C)		
CO_2_	5	150	62.76	74.10
N_2_	5	150	30.35	44.30
He	5	150	22.19	14.80

**Table 7:** Foaming properties of thermoplastic polyurethanes (TPU) according to saturation pressure.

Experimental Conditions			Number of Cells	Foaming Ratio (%)
Species of Gas	Pressure (MPa)	Temperature (°C)		
CO_2_	3	150	47.38	63.90
	4	150	54.59	68.50
	5	150	62.76	74.10

**Table 8:** Foaming properties of thermoplastic polyurethanes (TPU) according to foaming temperature.

Experimental Conditions			Number of Cells	Foaming Ratio (%)
Species of Gas	Pressure (MPa)	Temperature (°C)		
CO_2_	5	50	26.61	17.40
		100	42.36	49.40
		150	62.76	74.10
